# Body Image Perception, Eating Habits, and Nutritional Status of Female University Students: A Case of Makerere University, Uganda

**DOI:** 10.1155/jnme/7059171

**Published:** 2025-05-04

**Authors:** Bridget Ainembabazi, Agnes Nabubuya, Ivan Muzira Mukisa

**Affiliations:** Department of Food Technology and Nutrition, School of Food Technology, Nutrition and Bioengineering, College of Agricultural and Environmental Sciences, Makerere University, P.O. Box 7062, Kampala, Uganda

**Keywords:** body image, body image dissatisfaction, eating habits, female students, skipping meals, snacking, Uganda

## Abstract

**Background:** Body image perceptions among young female adults significantly influence their quality of life, nutritional status, and wellbeing. Positive body image is characterized by high self-esteem and accepting one's body as is. This study assessed body image perception, eating habits, and nutritional status of female university students.

**Methodology:** A cross-sectional survey was conducted among female students of Makerere University in Kampala, Uganda. Participants were recruited through convenience sampling. Anthropometry, body image perceptions, and eating habits were evaluated. Body image perceptions and eating habits were assessed using a questionnaire.

**Results:** Majority of the respondents (68%) had normal weight, 25% were overweight, 4% were underweight, and 3% were obese. About half (51%) of the respondents were dissatisfied with their body weight and shape while 49% felt fat and had a strong desire to lose weight. About 69% of the respondents skipped meals and 69% snacked at least once a day.

**Conclusion:** A big proportion of female students expressed dissatisfaction with their body image despite majority having normal weight status. Targeted mental health programs should be designed to help students deal with dissatisfaction and promote general wellbeing.

## 1. Introduction

The transition of young people from high school to university is often accompanied by significant lifestyle changes, particularly in diet and body image perceptions [1]. Regardless of gender, during young adulthood, individuals experience significant bodily changes that heighten awareness of physical characteristics and increase the likelihood of body image concerns [2].

Body image is a perception of one's physical appearance and the feelings that arise from it [1, 3]. These feelings and emotions resulting from their body measurements and shape influence their body satisfaction [3]. Body image dissatisfaction has been associated with a lower quality of life and is significant in the diagnosis of anorexia nervosa and bulimia nervosa [1, 3, 4], which can contribute to malnutrition. A study in England and Denmark revealed that 8.6% of the students perceived themselves as “too thin,” 37.7% perceived themselves as “just right,” and 53.7% perceived themselves as “too fat” [1].

Body image perception is affected by a combination of factors like media and society. The portrayal of unrealistic body ideals in media significantly contributes to body image dissatisfaction [5]. Media platforms, including advertising and social media, perpetuate narrow beauty standards, promoting body comparison, self-criticism, and dissatisfaction [1, 6]. Studies have revealed that females tend to have more elevated body image concerns than males and while they exist in all stages of life, they are significant in adolescence and adulthood [3, 5, 7].

Body image dissatisfaction is often linked to unhealthy eating behaviors such as restrictive eating or excessive energy intake, both of which are associated with a higher risk of overweight and obesity. During periods of restrictive eating, the body undergoes hormonal changes involved in appetite regulation and alterations in metabolism and energy expenditure [8]. These hormonal changes including reduced thyroid hormones and increased cortisol promote energy storage in the body. In addition, decreased leptin and insulin and increased ghrelin contribute to increased food intake. These hormonal changes do not only exist during the period of restrictive eating but also remain active long after initial weight reduction hence favoring weight gain [8]. Excessive energy intake from sugary and fatty foods, genetic predisposition, stress, depression, and weight gain, can all contribute to noncommunicable diseases (NCDs) such as diabetes and cardiovascular diseases.

Eating habits among individuals can be influenced by numerous factors such as body image, culture, and peer influence [9]. Dietary patterns that are quite common among young adults include snacking, usually on energy-dense foods; meal skipping, particularly breakfast or irregular meals; wide use of fast food; and low consumption of fruits and vegetables [10]. Some individuals, particularly females who are more conscious of their diet, deliberately skip breakfast due to increased concerns about body weight and appearance [10, 11]. These unhealthy eating behaviors can lead to undernourishment or overnourishment, resulting in increased susceptibility to overweight, obesity, and NCDs.

Overweight and obesity are characterized by excessive fat deposits that can impair health. Body mass index (BMI) is the primary tool used to diagnose overweight and obesity, defined by BMI ≥ 25 as overweight and BMI ≥ 30 as obesity for adults. Overweight and obesity are among the leading preventable causes of diabetes, cardiovascular diseases, and other comorbidities. According to a study carried out in Nigeria, the prevalence of overweight and obesity among female students was 21.3% [12]. Overweight and obesity are very likely to affect how people perceive their body image.

Body image concerns and their consequences were earlier thought to exist solely in the western world [7]. Studies exploring body image and eating habits have been carried out in African communities [13] and Ugandan adolescent and rural population [14, 15]. However, there is no study documenting the body image concerns and related eating habits in the female university population in Uganda. Body image dissatisfaction can result into unhealthy eating behaviors such as restrictive eating, excessive energy consumption, and meal skipping; therefore, understanding the relationship between body image perceptions and eating habits is essential. This study aimed to determine the body image perceptions, eating habits, and nutritional status of female students at Makerere University.

## 2. Methods

### 2.1. Study Design

A cross-sectional study was conducted at Makerere University, Kampala, Uganda from June to July 2022 using female students as the participants. Participants were chosen through convenience sampling, focusing on the three halls of residence for females followed by the random selection of lecture rooms within the colleges. Both qualitative and quantitative data on anthropometry, body image, and eating habits were collected.

The sample size (*n* = 275) was calculated using Cochran's sample size formula [16] with a 5.9% margin of error, 95% confidence interval, and proportion of 0.5 since no study had been conducted on this topic in Uganda at that time. The total sample size was distributed to ensure that female students from all halls of residence and colleges of study participated. Participants were selected by convenience sampling. Female students who were able to self-report, not visibly pregnant, and had no mobility impairments were eligible to take part in the study. This study aimed to assess the nutritional status, body image, and eating habits specifically in the Ugandan population; therefore, all study participants were Ugandan.

### 2.2. Data Collection

Participants self-reported their height and weight while measured values were obtained for 130 participants to assess reporting accuracy. Height was measured using a stadiometer (Seca, 213 1721009, GmbH & co., US design patent) and recorded in centimeters to one decimal place. Body weight was measured using a weighing scale in kilograms (Beurer GmbH Soflinger Str. 218 89077 Ulm, Germany) and recorded to one decimal place. Participants were weighed in bare feet and light clothes. Both self-reported and measured height and weight measures were used to calculate BMI and categorized as shown in Table 1.

(1) $$\begin{array}{c} \text{BMI}=\frac{\text{Weight}\left(\text{kg}\right)}{\text{Height}\left(m^{2}\right)}. \end{array}$$

Body image perception was assessed by asking participants questions that gauged how they felt about their bodies in the last 28 days. The Eating Disorders Examination Questionnaire (EDE-Q 6.0) shape and weight concern subscales were adopted for this study. Other subscales in the EDE-Q 6.0 include restraint and eating concern [18]. The Cronbach's alpha values were 0.93 for shape concern, 0.89 for weight concern 0.81 for restraint, and 0.89 for eating concern, indicating internal consistency and test reliability [19]. Participants were asked questions concerning their desire to lose weight, body shape, and weight dissatisfaction as well as feeling fat.

Snacking and skipping meals were the main eating habits that were assessed in this study. The questions were adopted from questionnaires used in similar studies on eating habits [10, 20]. Participants were asked whether they snacked and how often they snacked. They were also asked whether they skipped the main meals of the day, how often, and why they skipped meals.

### 2.3. Data Analysis

The data was analyzed using StataSE 15.0 software (StataCorp LLC 4905 Lakeway Drive College Station, Texas 77845 USA). Quantitative variables were presented as means and standard deviations while qualitative variables were presented as frequency and percentages. Descriptive means and standard deviation statistics were used to analyze eating habits and body image. Pearson's Chi-square test was used to determine whether there was a difference between the self-reported BMI and the actual measured BMI. A *p* value < 0.05 was considered to be statistically significant at 5% level of significance.

### 2.4. Ethical Considerations

All participants were provided with detailed information about the study, its purpose, procedures and their rights as participants. Participation was voluntary. Every participant was required to sign consent forms and no incentives were given. All data was anonymous and confidential.

## 3. Results and Discussion

### 3.1. Age of the Respondents

The respondents were aged between 18 and 35 years, with the majority (91.6%) being 18–23 years (Table 2). This trend may be attributed to the fact that most graduates from secondary school seek admission to university shortly after graduation and thus fall within the same age group. This is similar to the findings of Omage and Omuemu [10], where the majority (41.0%) of the study population belonged to the 19–21 years age group. Similarly, in another study [21], the modal age group was 19–24 years, comprising 57.9% of the participants.

### 3.2. Body Image Perception and Nutritional Status of Female Students

#### 3.2.1. Appearance and Body Dissatisfaction

**Figure 1:**
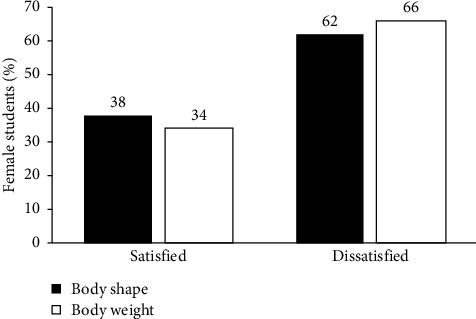
Proportion of respondents who were dissatisfied with their body shape and weight (*n* = 275).

Body dissatisfaction is the difference between current and desired body attributes [22]. The majority of the respondents were dissatisfied with their body weight (66%) and shape (62%) (Figure 1). Fifty-one percent of respondents were dissatisfied with both shape and weight. Body weight and shape dissatisfaction are likely to coexist in the same individual because body weight and shape changes occur concurrently supported by a statistically significant positive correlation between body weight dissatisfaction and body shape dissatisfaction (*r*_*s*_ = 0.5367, *p* < 0.001, Table 3).

The results of this study are in agreement with those reported by other researchers who found varying percentages of respondents who were dissatisfied with their body shape; 48% for undergraduate female students in Malaysia [5] and 91% for college students in the United States. The variation in proportions might be attributed to the societal differences caused by the idealization of the slim body among the different populations [1]. Body dissatisfaction is of great concern because it has been related to disordered eating behaviors like restraint and poorer physical and mental health, which all lead to poor quality of life [23].

#### 3.2.2. Perception of Body Size and Desire to Lose Weight

**Figure 2:**
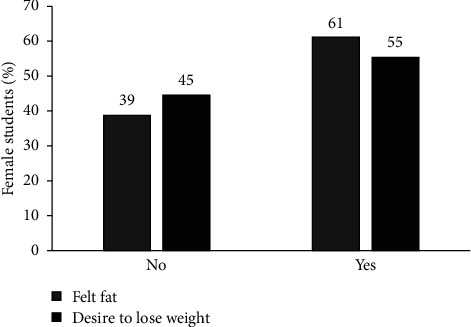
Proportion of respondents who had the desire to lose weight and felt fat (*n* = 275).

The majority of the respondents (61%) felt fat while 55% had a strong desire to lose weight (Figure 2). These findings might suggest that societal idealization of certain body types, particularly slim body types, accompanied by cultural and peer pressures, are a deeply ingrained part of the female population. This study was conducted in the context of the coronavirus disease 2019 (COVID-19); therefore, it is important to consider that factors related to the COVID-19 scenario affected these results. de Castro et al. [24] investigated body dissatisfaction during the pandemic which revealed an increase in sedentary behaviors, food intake, and sleep as well as a reduction in behaviors related to health and quality of life as body dissatisfaction intensifiers. Continued exposure to the internet and social media creates pressure for people to look a certain way, resulting into increased desire to lose weight and achieve slimmer bodies [6, 7]. The students also mentioned that bigger body sizes looked bad and unhealthy, which may explain why the majority of the respondents desired to lose weight. Fear of obesity-related illnesses might also contribute to the desire to lose weight among the female students. A study conducted among adults in South Africa revealed that some participants believed that being overweight causes diseases [25]. A study by [1] revealed that 27.4% of the respondents had inaccurate body image perception resulting in a strong desire to modify their bodies by engaging in more physical activity. Continuous obsession about idealized body appearances can result into dangerous compensatory behavior.

#### 3.2.3. Accuracy of Self-Reported Height and Weight

**Figure 3:**
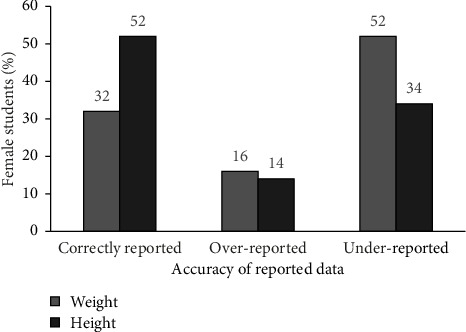
Accuracy of self-reporting anthropometric measurements (*N* = 130).

This study was interested in determining the accuracy of self-reported height and weight measurements and how effective these would be for evaluating the nutritional status of university students.  Figure 3 shows the proportion of respondents who correctly reported, over-reported, and underreported their weight and height measurements. About half of the respondents (52%) underreported their weight (within 1.5 kg) and height was also correctly reported for half (52%) of the respondents (within 1.4 cm). These results suggest that while self-reported height and weight measurements can be slightly underreported, they are reliable when actual anthropometry measurements cannot be taken. Underreporting weight measurements may be caused by the social desirability to have lower body weight [26]. The errors in self-reported measurements, however, might also be attributed to the few opportunities for respondents to regularly measure their height and weight, as well as quick fluctuations in weight. The findings of this study are similar to those of Bowring et al. [26] who concluded that weight is usually underreported. These results are also similar to those of a study by Lipsky et al. [27], which showed that females were more likely to underreport weight compared with height. However, other studies prove that self-reported anthropometry is reliable [28].

#### 3.2.4. Nutritional Status of Female Students

**Figure 4:**
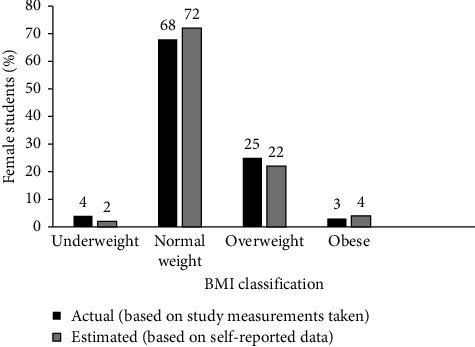
Nutritional status of respondents based on BMI.

Figure 4 shows the proportion of respondents in the various BMI categories determined using self-reported and measured height and weight. Based on actual BMI calculations, 68% of the respondents had normal weight, which is slightly less than the proportion of normal weight respondents based on estimated BMI calculation (72%). Majority (68%) of the respondents were normal weight with respect to BMI calculated from measured weight and height, 25% were overweight, 4% were underweight, and 3% were obese. These findings suggest that BMI from self-reported anthropometry could be reliably used to estimate the nutritional status of a population. However, despite the similarities in the proportion of respondents in the different categories, there was a statistically significant difference between self-reported and actual BMI (Pearson chi^2^ = 95.5344 and *P*_*r*_ = 0.000). It is important to note that the use of self-reported height and weight measurements is applicable to educated people and, therefore, might not apply to all populations [29].

The use of BMI provides critical insights into the nutritional status of the population. The existence of underweight (4%), overweight (25%), and obesity (3%) in the sample indicates a double burden of malnutrition, which is a global concern. The double burden of malnutrition indicates that the Sustainable Development Goals SDG2 and SDG3 of ending all forms of malnutrition and ensuring healthy lifestyles and wellbeing for all people of all ages have not yet been achieved [30]. However, according to the World Health Organization [31], 3%–5% of healthy adult population have BMI < 18.5, indicating that prevalence of 4% is not of public health significance. The presence of underweight (4%) and obesity (3%) at low percentages are not a cause for concern but are important considerations for policy planning. The normal BMI range is considered optimal for long-term health and wellbeing. Public health initiatives and policies that promote healthy lifestyles can help sustain this positive trend and prevent shifts toward underweight and obesity categories.

### 3.3. Eating Habits Among Female Students

#### 3.3.1. Meal Skipping Habits

About 7 in 10 respondents (69%) said they skipped meals at least once a week in the past 28 days (results not shown). These results were similar to a study carried out in Malaysia during the COVID-19 era, where about 52.4% of respondents skipped at least one meal a day [11]. About 63% of the respondents skipped meals every 1–2 days in a week to lose weight (Table 4).

There was no significant correlation between the desire to lose weight and meal skipping frequency (*r*_*s*_ = 0.0361, *p* = 0.5501, Table 3), suggesting that majority of the students skipped meals due to other reasons rather than a desire to lose weight. Some of the other reasons may include frequent snacking, limited time, out of habit, and financial constraints [32–34]. Makerere University does not provide free meals to students and they are expected to buy their own meals; some privately while the government sponsored students are given an allowance of about UGX. 4400 (1.19 USD) per day. This money provided is insufficient to cover 3 meals a day because on average the cheapest meal costs about UGX. 3000 (0.81 USD); therefore, they also have to privately buy some of their meals. This occurrence is similarly what was reported by Otemuyiwa and Adewusi [35], who noted that most university students in Nigerian universities skipped meals because they did not have enough money to spend on both food and other basic and academic requirements. Other studies by Ogundele et al. [36] and Rudolph et al. [37] also showed that food cost had a significant effect on eating habits among African university students and meal skipping was a food insecurity coping strategy.

A study by Ainomugisha [9] reported that students skipped meals to lose or maintain weight. Skipping meals as a weight loss strategy is a popular form of dieting aimed at reducing caloric intake to control body weight [1]. The correlation between body weight dissatisfaction and meal skipping frequency was close to zero, indicating a very weak linear relationship (*r*_*s*_ = 0.0045, *p* = 0.9413, Table 3). In a systematic review by Pendergast et al. [32], nine of the ten studies reported that time had the greatest perceived influence on meal skipping when it was ranked against other significant predictors of meal skipping in young adults. Young adulthood is characterized by transitions like moving out of the family home, starting a career, and further education. Earlier research also showed that time scarcity had a negative effect on a range of eating habits [38].

Lack of time may be due to a lack of prioritization of meal times and good eating habits, evidence that an individual's time perspective is a powerful influence on their behavior. The lack of time is always self-reported by individuals and can be interpreted in various ways. Pendergast et al. [32] suggested that “time” may have been attributed to food shopping, preparation, cooking time, or eating time and may have been interpreted differently by individuals. Since individual characteristics underlie people's perception of time, methods in this study were not detailed enough to provide a conclusive association with meal skipping.

Several respondents frequently said that *“lunch is a mindset.”* Another respondent remarked that she rarely eats breakfast during the week because she is always late for class, suggesting that it is a habit. A study by Pendergast et al. [33] in Australia reported that reasons for skipping meals varied with the different meals in the day. Smoking was positively associated with skipping breakfast while limited time was positively associated with skipping breakfast and lunch. These findings are similar to those of Seedat and Pillay [39] who concluded that students in South Africa skipped breakfast because of time constraints and lack of appetite. In America, University students who skipped meals reported that they had to work during after-school hours; therefore, they had neither the money nor the time to shop and prepare for all meals [40]. They also reported that students who lived away from home skipped more meals than those who commuted from home to school. Staying away from home is a common factor that is leading to skipping meals among university students.

#### 3.3.2. Snacking Habits Among Female Students

**Figure 5:**
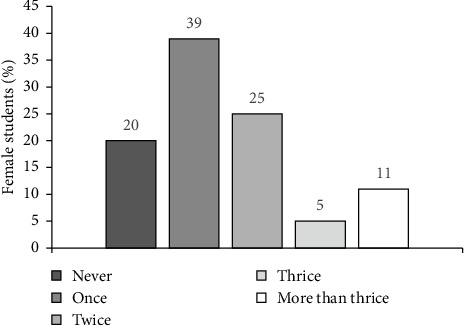
Frequency of snacking in a day by female students (*n* = 275).

A majority of the respondents (80%) reported engaging in snacking (Figure 5). Of these, 39% snacked once a day, 25% snacked twice a day, and 11% snacked four times a day. This proportion is much higher than that of a study carried out in Coastal South India, where 54.3% of the participants snacked [41]. These findings are similar to those of Pung et al. [11], who reported that 94.6% of respondents snacked between meals.

The high rate of snacking can be attributed to the high level of meal skipping evidenced by the statistically significant positive correlation between the frequency of snacking and skipping meals (*r*_*s*_ = 0.2529, *p* < 0.001, Table 3). Students frequently skipped meals due to time constraints, opting for easily accessible snacks as a quicker alternative. This can also be explained by the poor prioritization of meal times. The correlation between body weight dissatisfaction and snacking was close to zero (*r*_*s*_ = −0.0810, *p* = 0.1811, Table 3). These findings suggest that the students' snacking frequency was influenced by other factors more strongly than body weight dissatisfaction alone.

### 3.4. Strengths of This Study

The study utilized the EDE-Q 6.0 which is a validated questionnaire, making the findings reliable. Since no similar study had been conducted in Uganda at that time, data from this study can be used as a baseline to inform future studies.

### 3.5. Limitations of This Study

The use of convenience sampling limits the generalizability of the findings to the broader population of female students. Assessing eating habits was completely reliant on memory which exposed the research to respondent bias. Actual anthropometric measurements were not taken for all study participants due to time constraints and, therefore, the sample was smaller in regards to some conclusions of the study. Limited data on sociodemographic characteristics were collected.

## 4. Conclusions

This study documented a double burden of malnutrition among the female students in Makerere University. Majority of the female students were dissatisfied with their body image despite most of them falling within the normal weight category. A significant proportion of the students engaged in unhealthy eating behaviors such as skipping meals and snacking, which were influenced by both financial and time constraints rather than a desire to lose weight. Although body dissatisfaction was prevalent, it did not show a strong correlation with meal skipping, suggesting that other factors, such as socioeconomic factors may play a more prominent role in shaping eating habits.

Future studies should investigate the effectiveness of targeted interventions such as mental health programs in promoting healthier eating habits and improving body image perceptions among university students. More detailed qualitative research, such as focus group discussions, could help to understand the underlying social and psychological factors influencing healthy eating habits.

## Figures and Tables

**Table 1:** BMI classification.

BMI	BMI category
< 18.5	Underweight
18.5–24.9	Normal
25–29.9	Overweight
≥ 30	Obese

*Note:* Source: World Health Organization [17].

**Table 2:** Proportion of respondents in the age categories and colleges of study (*N* = 275).

	Categories	Mean	SD	Percentage (%)
Age of respondents	18–23 years	21.58	1.00	91.6
	24–29 years	24.94	1.47	6.9
	30–35 years	35	—	1.5

**Table 3:** Pairwise correlations between meal skipping, weight and shape dissatisfaction, feeling fat, desire to lose weight, snacking frequency, and frequency of skipping meals for female students.

	Meal skipping	Weight dissatisfaction	Shape dissatisfaction	Frequency of skipping meals	Frequency of snacking	Felt fat	Desire to lose weight
Meal skipping	1						
Weight dissatisfaction	0.0324 (0.5930)	1					
Shape dissatisfaction	−0.0866 (0.0735)	0.5367 (0.0000)^∗^	1				
Frequency of skipping meals	0.7298 (0.0000)^∗^	0.0045 (0.9413)	−0.0358 (0.5562)	1			
Frequency of snacking	0.2503 (0.000)^∗^	−0.0810 (0.1811)	−0.1926 (0.0014)^∗^	0.2529 (0.0000)^∗^	1		
Felt fat	−0.0121 (0.8416)	0.2103 (0.0004)	0.2704 (0.0000)	0.0164 (0.7859)	0.0580 (0.3383)	1	
Desire to lose weight	0.0092 (0.8785)	0.2892 (0.0000)^∗^	0.2657 (0.0000)^∗^	0.0361 (0.5501)	0.0002 (0.9977)	0.6770 (0.0000)^∗^	1

*Note:* Values with ^∗^ are significant at *p* < 5%.

**Table 4:** Reasons for skipping meals and the meal skipping frequency in a week (*N* = 275).

Frequency of skipping meals	Reason for skipping meals			
	Limited time (%)	To lose weight (%)	Lack of money (%)	Lack of appetite (%)
1–2 days	59	63	43	54
3–5 days	13	15	24	21
Everyday	28	22	33	25

## Data Availability

Data supporting the findings of this study are available on request from the corresponding author.
